# Eye-in-Hand Robotic Arm Gripping System Based on Machine Learning and State Delay Optimization †

**DOI:** 10.3390/s23031076

**Published:** 2023-01-17

**Authors:** Chin-Sheng Chen, Nien-Tsu Hu

**Affiliations:** Graduate Institute of Automation Technology, National Taipei University of Technology, Taipei 10608, Taiwan

**Keywords:** point cloud, neural network, arm control, object grabbing, state delays

## Abstract

This research focused on using RGB-D images and modifying an existing machine learning network architecture to generate predictions of the location of successfully grasped objects and to optimize the control system for state delays. A five-finger gripper designed to mimic the human palm was tested to demonstrate that it can perform more delicate missions than many two- or three-finger grippers. Experiments were conducted using the 6-DOF robot arm with the five-finger and two-finger grippers to perform at least 100 actual machine grasps, and compared to the results of other studies. Additionally, we investigated state time delays and proposed a control method for a robot manipulator. Many studies on time-delay systems have been conducted, but most focus on input and output delays. One reason for this emphasis is that input and output delays are the most commonly occurring delays in physical or electronic systems. An additional reason is that state delays increase the complexity of the overall control system. Finally, it was demonstrated that our network can perform as well as a deep network architecture with little training data and omitting steps, such as posture evaluation, and when combined with the hardware advantages of the five-finger gripper, it can produce an automated system with a gripping success rate of over 90%. This paper is an extended study of the conference paper.

## 1. Introduction

The COVID-19 pandemic means that there is a greater demand for automated production. To avoid a reduction in production capacity due to insufficient manpower, robotic arms with automatic control systems must perform more complex and more varied tasks.

Nowadays, robot arms for pick-and-place and assembly tasks have matured [1,2,3,4,5,6,7,8], and many factory robot arms now use two- or three-finger grippers for tasks, and many research papers have been conducted in this direction or for experiments [9,10,11,12]. Some use algorithms for control [13] and others use visual images and machine learning to allow robotic arms to perform tasks [14,15]. Other studies convert images into point clouds that use depth distances for better task execution [16,17,18,19,20,21,22,23,24]. For example, the study in [25] combines object recognition by Mobile-DasNet and point cloud analysis to generate the coordinates of the arm endpoints for the apple picking task.

However, if the factory is to become more automated, the robot arm needs to be able to perform more complex movements. On this premise, the original two- and three-finger grippers may not be able to complete more delicate or complex movements, so many people began to develop and use other types of grippers to overcome these shortcomings, including the five-finger grippers. Although the five-finger grippers have a larger gripping range, higher flexibility and fault tolerance, because of their complex structure, many control systems that use two- or three-finger grippers, as to derive the gripping attitude may not be compatible with five fingers.

There have been some studies using five-finger graspers, but most of them studied hardware or other aspects [26,27,28,29]. The studies used for application have used a virtual space as a result of the study [30,31] or used algorithms for gripping pose prediction [32,33].

This paper is an extended study of the original conference paper [1]. We extend the original conference paper on the effects of time delay for the robot manipulator. We investigate state time delays and propose a control method for a robot manipulator. Many studies on time-delay systems have been conducted, but most focus on input and output delays. One reason for this emphasis is that input and output delays are the most commonly occurring delays in physical or electronic systems. An additional reason is that state delays increase the complexity of the overall control system. In this paper, we use the qbSoftHand five-finger grasper in combination with an RGB-D visual recognition system and modify the proposed mechanical learning network by omitting the pose evaluation part so that the arm can automatically move to the target location and accomplish the task of grasping the target object to the placement area. The state delay of the control system is also optimized to enable the system to operate efficiently. Experiments were conducted with objects that were considered difficult to grasp with a two- or three-finger grasper to demonstrate the superiority of the grasping method and five fingers.

## 2. Control System Architecture

**Figure 1:**
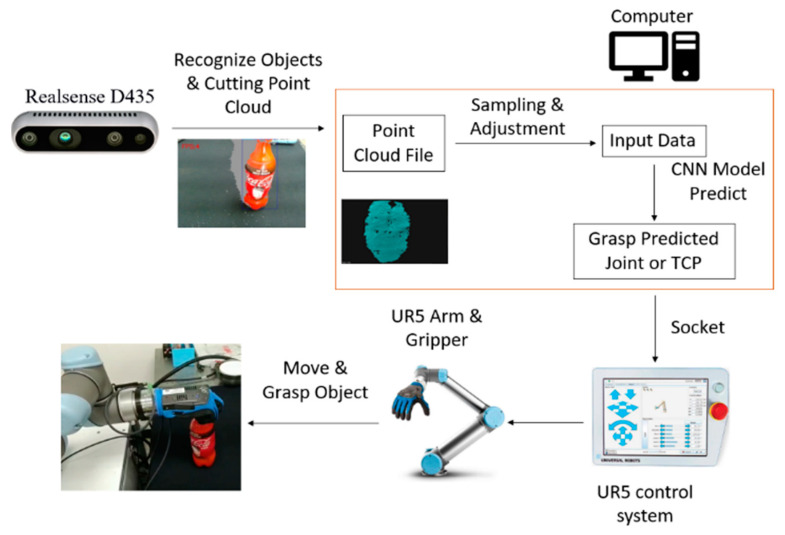
The working principle of the gripping robot.

The hardware for this study is a UR5 six-degree-of-freedom arm, qbSoftHand five-finger gripper, HIWIN XEG-32 gripper and a RealSense D435 camera. The system architecture is shown in Figure 1. Object recognition is achieved using Yolo [34,35]. The point cloud for the object is generated after sampling and adjustment. The data are used as input data for an AI model that predicts the gripping posture for the arm and sends this information to the arm to perform the gripping action.

The pose that is predicted by the network is sent to the UR5 control system via the socket library of the network cable. The current joint angle, the tool center point (TCP) and the analog and digital signals for the arm are also obtained in this way.

A successful grasping action is achieved if the arm moves to the target location, picks up the object and moves it to the target position without dropping the object.

## 3. Point Cloud Data and Machine Learning Architecture

### 3.1. Convolutional Neural Network (CNN) Architecture

**Figure 2:**
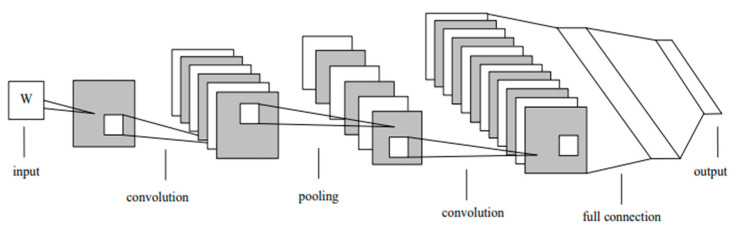
The network architecture of CNN [36].

The architecture of the CNN is shown in Figure 2. This section describes the hidden layer (convolutional layer). 

The convolutional layer is the core of the CNN [36]. When the image is input into the convolution layer, it performs convolutional operations using the convolutional kernel. The formula is:

(1) $$X_{j}^{l}=f(\sum_{i\in Pj}X_{i}^{l-1}*k_{ij}^{l}+b_{j}^{l})$$

where $f(\sum_{i\in Pj}X_{i}^{l-1}*k_{ij}^{l}+b_{j}^{l})$ is a tanh function, $P_{j}$ is a local receptive field, $X_{i}^{l-1}$ is the value of the l-1 feature on the *i* window, (i,j) is the position on the first floor, $k_{ij}^{l}$ and $b_{j}^{l}$ are the respective weights of the convolution kernel and the offset of the feature. More details can be found in [37]. 

The input data for this study are not images, but numerical values, so the convolution is a one-dimensional convolution. Differences between this and a two-dimensional convolution are described in Section 3.3.

**Figure 3:**
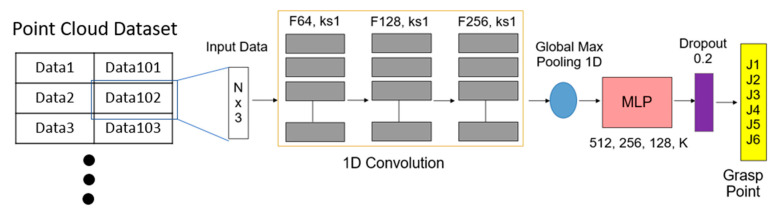
Grasping the estimated CNN network architecture. (N: the number of “ points” in input data; F: filters, ks: kernel size; K: output number).

The AI network for this study consists of three one-dimensional convolutional layers, a pooling layer and a multi-layer perceptron [25]. The point cloud data are input and the position at which the object is grasped is predicted. This information is transmitted to the UR5 arm control system. The network architecture using the CNN is shown in Figure 3.

### 3.2. Min-Pnet Architecture

**Figure 4:**
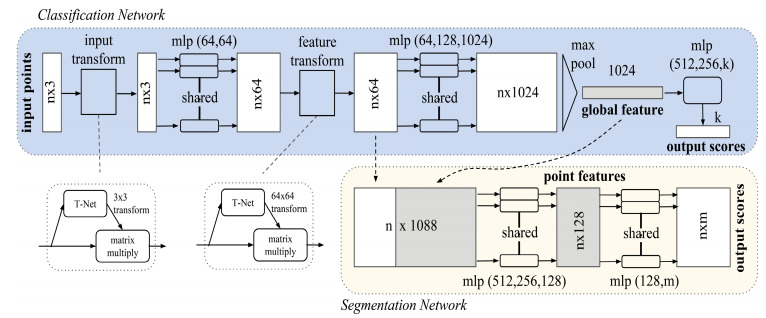
PointNet’s network architecture [37].

Min-Pnet is a network designed according to the architecture of PointNet [37] (see Figure 4) In PointNet research, it is mentioned that many studies transform the input data into regular 3D voxel grids or collections of multi-angle images, generating large amounts of unnecessary data and destroying the natural invariance of the original data. This study uses point clouds directly to avoid these problems and to make it easier to learn.

In order to protect the network itself from point cloud disorder, PointNet uses its “feature extraction layer” to convert disordered point cloud data into 1024-dimensional features to perform subsequent tasks.

The feature extraction layer is to transform the input points into features by assembling the input data into a canonical space and using T-net to predict an affine transformation matrix. The partial and global feature extraction of the point cloud is performed without affecting the correlation and invariance of the points.

**Figure 5:**
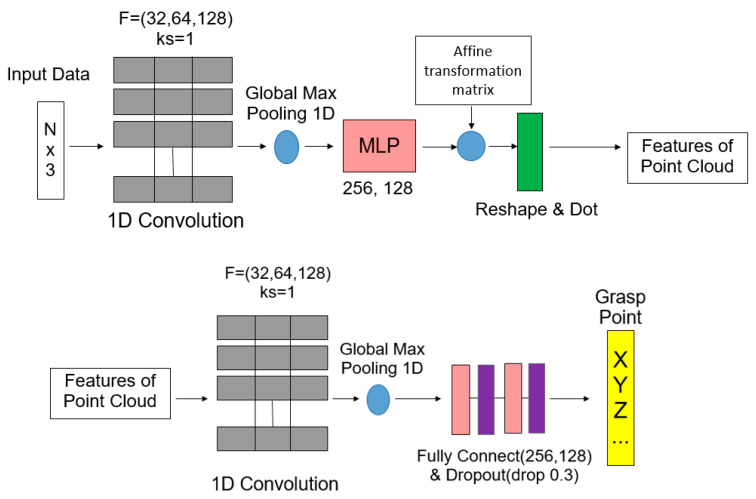
Min-Pnet architecture (N: the number of “points” in input data; F: filters; ks: kernel size).

In this study, the Min-Pnet (see Figure 5) uses one feature extraction layer instead of two to reduce the training time and to avoid overfitting. The original 2D conv part was changed to 1D conv. This is because our input data size is different from the original setting of Pointnet, which causes problems in the matrix multiply part of the network.

### 3.3. Input Point Cloud Data

**Figure 6:**
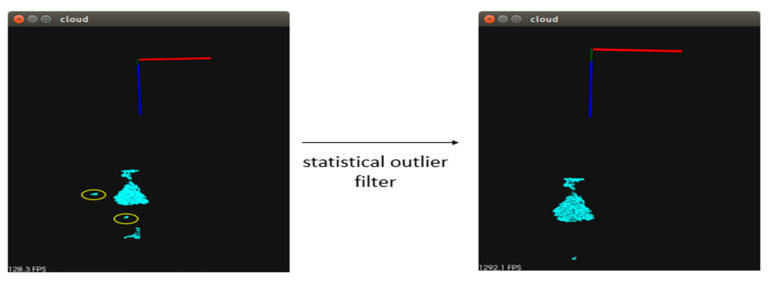
Schematic diagram of the statistical outlier filter. The yellow circle is the noise point removed.

To ensure that the point clouds are reliable and not easily affected by the external environment, a PCL [38] chopbox and the Yolo Bounding Box are used to remove most of the unwanted point clouds (walls and desktops; see Figure 6).

**Figure 7:**
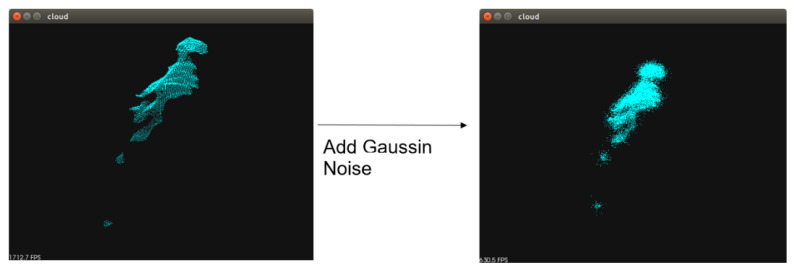
Point cloud sampling process.

To increase the amount of training data and to ensure that the model resists manipulation in response to slight errors that are caused by the hardware, Gaussian noise is used (Figure 7.)

**Figure 8:**
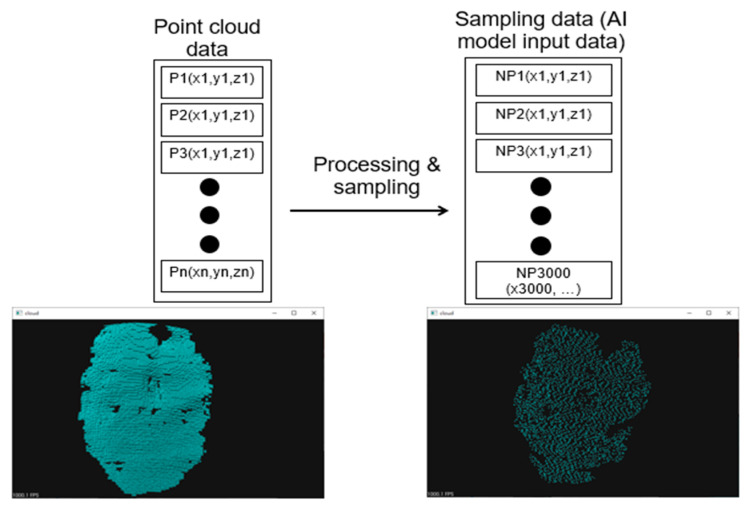
Point cloud sampling process.

To ensure that the input data are the same size, the point clouds for these objects are sampled and processed. The processed input data are a 3000 × 3 (N × 3 in Figure 8) matrix of the XYZ coordinates of the points (Figure 7). These data correspond to grasping point data, including the angle of each joint and a coordinate system based on UR5. These data are the input for the AI network and the prediction.

**Figure 9:**
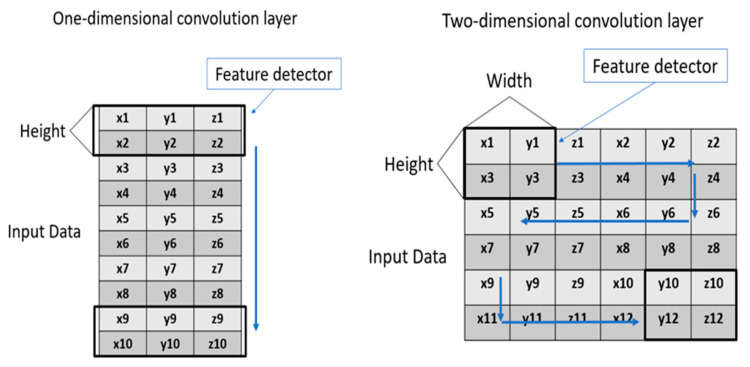
Illustration of 1D and 2D convolution works: the point cloud data for this study are used as demonstration.

A one-dimensional convolution layer (1D conv) is used for convolution and feature extraction from 1D data. Most AI frameworks that are used for object recognition or for grasp prediction, which use images, use a two-dimensional convolution layer (2D conv). The differences between these systems are shown in the Figure 9.

In the CNN network, 1D conv directly extracts features from the input point cloud, and after processing with the maximum pooling layer (Max Pooling), it finally inputs the multi-layer perceptron (MLP) composed of several fully connected layers to obtain the output of the final predicted grasp position. This study uses the position of the object in space to predict the grasping position of the gripper so a 1D conv is used to extract the main features. After we sample the point cloud data, it is input to the model as a network, and the model outputs the predicted grasp posture coordinates or joint angles.

A 1D conv is initially used for natural language and data analysis and can also be used to analyze point clouds in data format. It is less computationally intensive and requires a shorter training time than a 2D conv. A 2D conv cannot be used because the point cloud data are not continuous (Figure 9), so the extracted features cannot be applied, which affects the prediction results.

### 3.4. Pooling Layer

**Figure 10:**
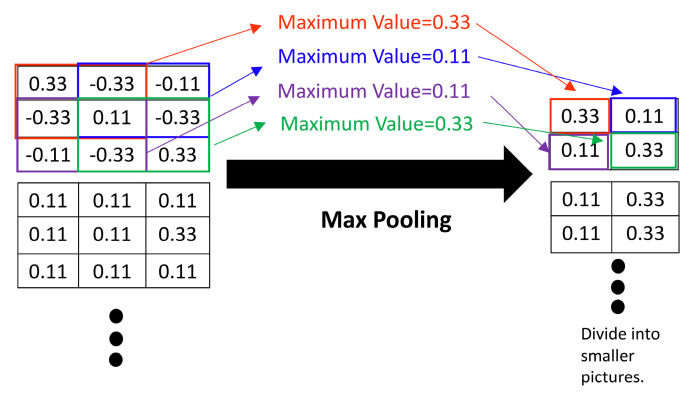
Operation of the Max Pooling layer.

The pooling layer reduces the dimensions of features and filters redundant features to reduce the computational burden and increase the generalization of the network. The pooling layer uses the maximum pooling (Max Pooling; see Figure 10) and mean pooling (Average pooling). The pooling process is expressed as:

(2) $$X_{j}^{i}=f(pool(X_{i}^{l-1})+b_{j}^{l})$$

where $X_{i}^{l-1}$ is the value of the *i*th window in the layer l-1 input feature, $b_{j}^{l}$ is the offset for the *j*th window in layer l and pool represents the sampling function.

### 3.5. Multi-Layer Perceptron (MLP)

Multi-layer perceptron uses several fully connected layers. Neurons in the full connection layer are connected to each other in the previous layer. The formula is:

(3) $$X^{l}=f(u^{l}).u^{l}=W^{l}X^{l-1}+b^{l}$$

where $f(u^{l})$ is the activation function, $W^{l}$ is the weight of layers l-1 to 1, $b^{l}$ is the offset for Layer 1, and $X^{l-1}$ is the output feature of Layer l-1.

The last fully connected layer outputs six parameters that are used to predict joint angles or TCP coordinates. To avoid overfitting and prediction errors in the AI model, an exit layer is inserted before the final output layer.

## 4. State Delays Using Digital Redesign

We present the principles of optimal digital redesign in this section. This system removes the following parts of the original transformation of a time-delay system to a delay-free system [39]:

(4) $$\dot{x}(t)=\;A\;x(t)+\;B\;u(t)$$

(5) $$y(t)=\;C\;x(t)\;,\;x(0)=\;x_{0}$$

The optimal quadratic state feedback control law is used to minimise the following performance cost function:

(6) $$J=\;\int_{\;0}^{\;\infty }\;\left\{{\left[\;Cx(t)-\;r(t)\;\right]}^{T}\;Q_{c}\;[Cx(t)-\;r(t)\;]+\;u_{}{}^{T}(t)R_{c}u(t)\;\right\}\;\;dt$$

where $Q_{c}\geq 0$, $R_{c}>0$. The following formula is obtained by optimising the controller:

(7) $$u(t)=\;-K_{c}x(t)+\;E_{c}r(t)$$

The entire closed loop system can then be expressed as

(8) $$\dot{x}(t)=\;(A-\;BK_{c})x(t)+\;BE_{c}r(t)$$

For m=p, we obtain

(9) $$K_{c}=\;R_{c}{}^{-1}B^{T}P$$

(10) $$E_{c}=\;-R_{c}{}^{-1}B^{T}{\left[{\left(A-\;BK_{c}\right)}^{-1}\right]}^{T}C^{T}Q_{c}$$

Here, P is the solution to the Riccati equation given below.

(11) $$A^{T}P+\;PA-\;PBR_{c}{}^{-1}B^{T}P+\;C^{T}Q_{c}C=\;0$$

**Figure 11:**
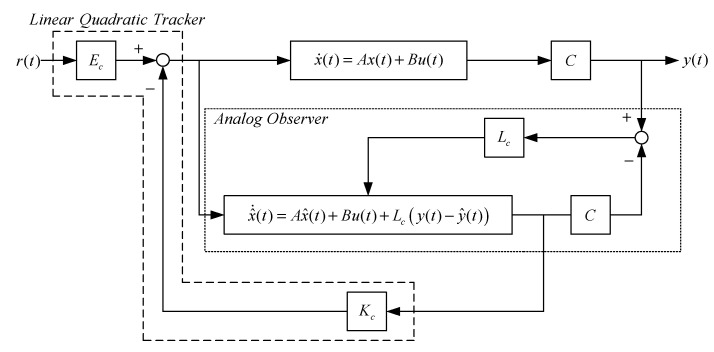
Linear-quadratic analogue tracker based on an observer.

The linear quadratic regulator (LQR) (Equation (6)) design characteristics make the resulting closed loop system stable. If the system state is unmeasurable, an observer must be designed to measure the system state. The linear observable continuous system (see Figure 11) that is described by the equation is shown below:

(12) $$\dot{\hat{x}}(t)=\;A\hat{x}(t)+\;Bu(t)+\;L_{c}[y(t)-\;C\hat{x}(t)]$$

where $\hat{x}(t)$ is the estimated state, and $L_{c}$ is the gain of the observer:

(13) $$L_{c}=P_{o}C^{T}R_{o}{}^{-1}$$

We apply digital redesign to the analogue controller (Equation (7)) to obtain a more practical digital controller. The operation of the discrete time state feedback controller is described by the following equation:

(14) $$u(kT_{s})=\;-K_{d}x(kT_{s})+\;E_{d}\;r^{*}(kT_{s})$$

where

(15) $$K_{d}=\;{\left(I+\;K_{c}H\right)}^{-1}K_{c}G$$

(16) $$E_{d}=\;{\left(I+\;K_{c}H\right)}^{-1}E_{c}$$

(17) $$r^{*}(kT_{s})=\;r(kT_{s}+\;T_{s})$$

(18) $$G=\;e^{AT_{s}}$$

(19) $$L_{d}=(G-I)A^{-1}L_{c}\;{\left(I+C(G-I)A^{-1}L_{c}\right)}^{-1}$$

**Figure 12:**
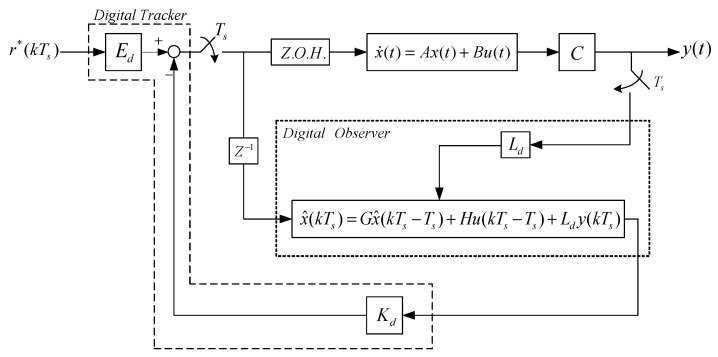
Predicted digital tracker and observer.

For the linear model of the sampling system, the digital tracker based on the observer and the observer are shown in Figure 12.

## 5. Results

**Figure 13:**
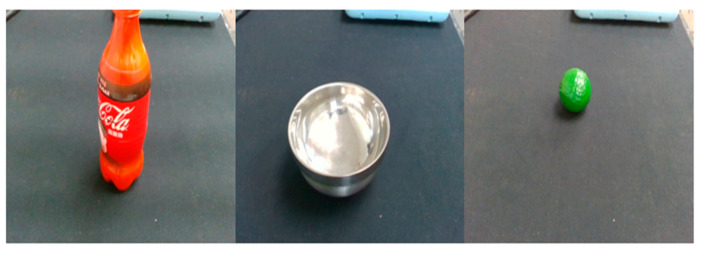
Three types of grasping objects for the experiment.

This study uses bottles, bowls and sports balls for the experiments (see Figure 13). The training parameters for the AI network are a batch size = 32 and an epoch = 10,000 and the loss parameter uses mean squared error (MSE). The selected optimizer is ‘adam’.

(20) $$MSE(y,\hat{y})=\frac{1}{n}\sum_{i=1}^{n}{\left(y_{i}-{\hat{y}}_{i}\right)}^{2}$$

$y_{i}-{\hat{y}}_{i}$ is error, n is number of data in Dataset. 

This study uses Python 3.7.7 and a Windows10 system environment for model training and a crawling test.

**Figure 14:**
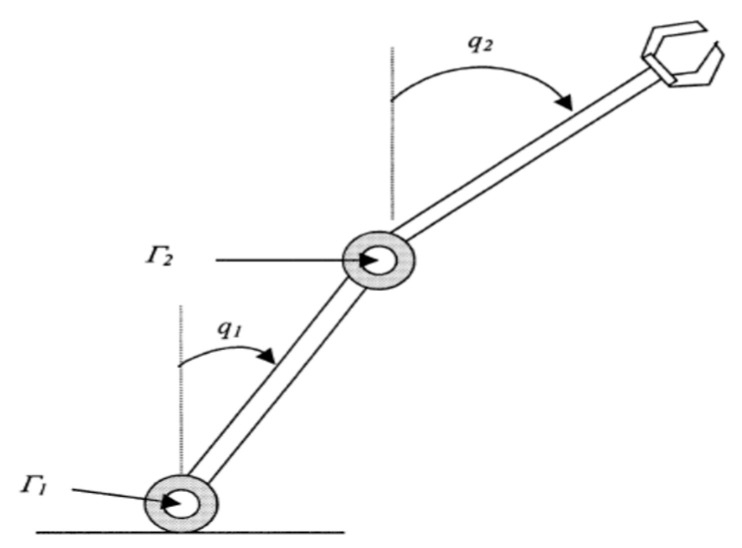
Two-link robot manipulator.

In order to verify that the proposed control method [40] can be applied in subsequent real-world tests and to collect data more easily, a simulator was used to design a model of a nonlinear MIMO robot manipulator, as shown in Figure 14.

### 5.1. The Test of the Robot Manipulator 

The dynamic equation of the two-link robot system is given below:

(21) $$M(q)\ddot{q}+C(q,\dot{q})\dot{q}+G(q)=\Gamma$$

where

$$\begin{array}{ll} M(q)=\left[\begin{array}{cc} (m_{1}+m_{2})l_{1}^{2} & m_{2}l_{1}l_{2}(s_{1}s_{2}+c_{1}c_{2})\\ m_{2}l_{1}l_{2}(s_{1}s_{2}+c_{1}c_{2}) & m_{2}l_{2}^{2} \end{array}\right] & ,\;C(q,\dot{q})=m_{2}l_{1}l_{2}(c_{1}s_{2}-s_{1}c_{2})\left[\begin{array}{cc} 0 & -{\dot{q}}_{2}\\ -{\dot{q}}_{1} & 0 \end{array}\right]\\ G(q)=\left[\begin{array}{c} -(m_{1}+m_{2})l_{1}g_{r}s_{1}\\ -m_{2}l_{2}g_{r}s_{2} \end{array}\right] \end{array}$$

and $q={\left[\begin{array}{cc} q_{1} & q_{2} \end{array}\right]}^{T}$, where $q_{1}$ and $q_{2}$ are angular positions, M(q) is the moment of inertia, $C(q,\dot{q})$ includes the Coriolis and centripetal forces, G(q) is the gravitational force, and Γ is the applied torque vector. Here, we use the short-hand notations $s_{i}=\sin(q_{i})$ and $c_{i}=\cos(q_{i})$. The nominal parameters of the system are as follows: the link masses are $m_{1}=5\;\mathrm{kg}$ and $m_{2}=2.5\;\mathrm{kg}$, the lengths are $l_{1}=l_{2}=0.5\;m$, and the gravitational acceleration is $g_{r}=9.81{\;\mathrm{ms}}^{-2}$. Then, (30) can be rewritten in the following form:

(22) $$\ddot{q}=M^{-1}(q)(\Gamma -C(q,\dot{q})\dot{q}-G(q))$$

Let x and f(x) represent the state of the system and a nonlinear function of the state x, respectively. The following notation is used:

$$x(t)\equiv {\left[x_{1}\;x_{2}\;x_{3}\;x_{4}\right]}^{T}={\left[q_{1}\;{\dot{q}}_{1}\;q_{2}\;{\dot{q}}_{2}\right]}^{T},\;f(x(t))\equiv {\left[f_{1}\;f_{2}\;f_{3}\;f_{4}\right]}^{T},$$

where

$f_{1}=x_{2}$, $f_{3}=x_{4}$, and ${\left[f_{2}\;f_{4}\right]}^{T}=M^{-1}(-C{\left[x_{2}\;x_{4}\right]}^{T}-G)$. Let u≡Γ, where $\Gamma ={\left[\begin{array}{cc} \Gamma_{1} & \Gamma_{2} \end{array}\right]}^{T}$.

The inverse of the matrix M is calculated as $M^{-1}=\left[\begin{array}{cc} p_{11} & p_{12}\\ p_{21} & p_{22} \end{array}\right]$, such that $g(x(t))={\left[\begin{array}{cccc} 0 & p_{11} & 0 & p_{21}\\ 0 & p_{12} & 0 & p_{22} \end{array}\right]}^{T}$.

The dynamic equation of the two-link robot system can, thus, be reformulated as follows:

(23) $$\dot{x}(t)=f(x(t))+g(x(t))u(t-\tau_{i})$$

$$y(t)=Cx(t-\tau_{o})$$

where $C=\left[\begin{array}{c} \begin{array}{cccc} 1 & 0 & 0 & 0 \end{array}\\ \begin{array}{cccc} 0 & 0 & 1 & 0 \end{array} \end{array}\right]$ and the initial condition is $x(0)={\left[\begin{array}{cccc} 0 & 0 & 0 & 0 \end{array}\right]}^{T}$.

**Figure 15:**
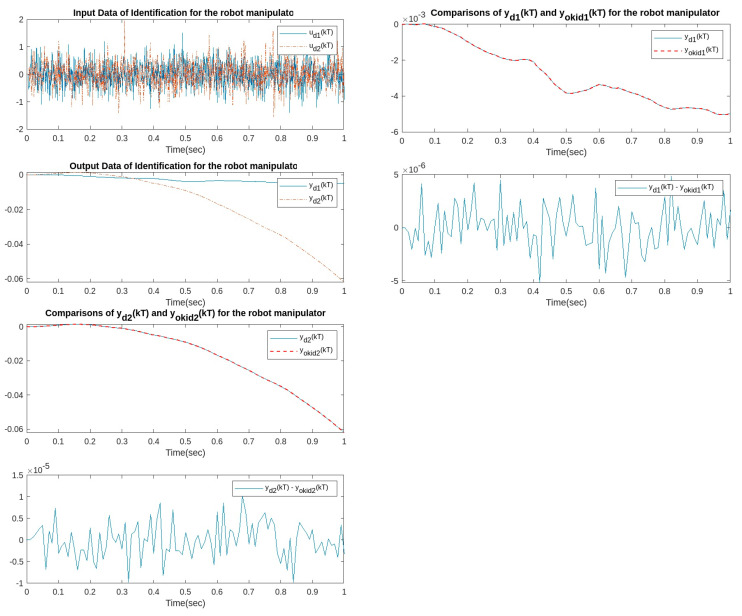
The comparison between the output of the identified equivalent linear system and the original nonlinear system.

First, OKID is applied to convert the nonlinear system (Equation (23)) to an equivalent linear system. The system (Equation (23)) is injected with white noise $u\left(t\right)=\left[\begin{array}{cc} u_{1}\left(t\right) & u_{2}\left(t\right) \end{array}\right]$ with a zero mean and covariance $diag\left(\mathrm{cov}\left(u_{1,2}(t)\right)=\left[\begin{array}{cc} 0.2 & 0.2 \end{array}\right]\right)$ at the sampling time T=0.01 s. Figure 15 shows that the error between the output of the identified equivalent linear system and the original nonlinear system (Equation (23)) can be controlled to within $10^{-6}\sim 10^{-5}$.

**Figure 16:**
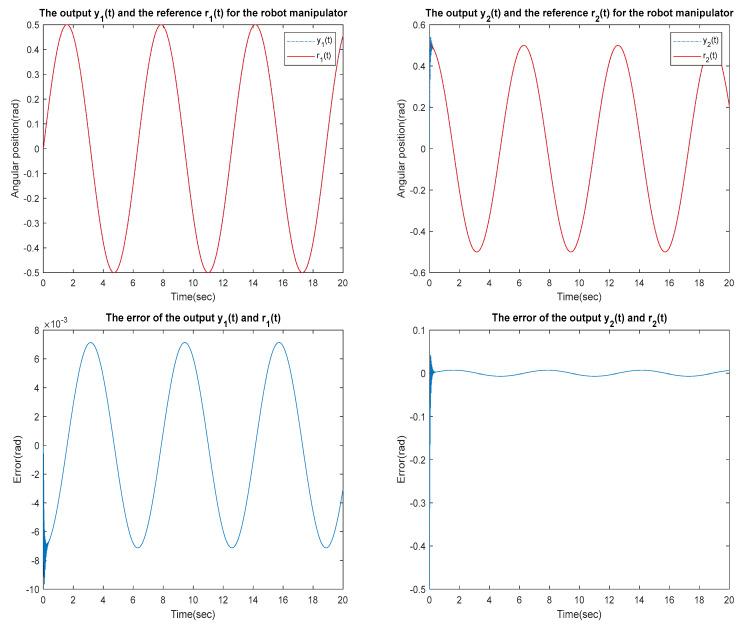
TYPE 1. Case 1: for the ability to suppress state delay.

**Figure 17:**
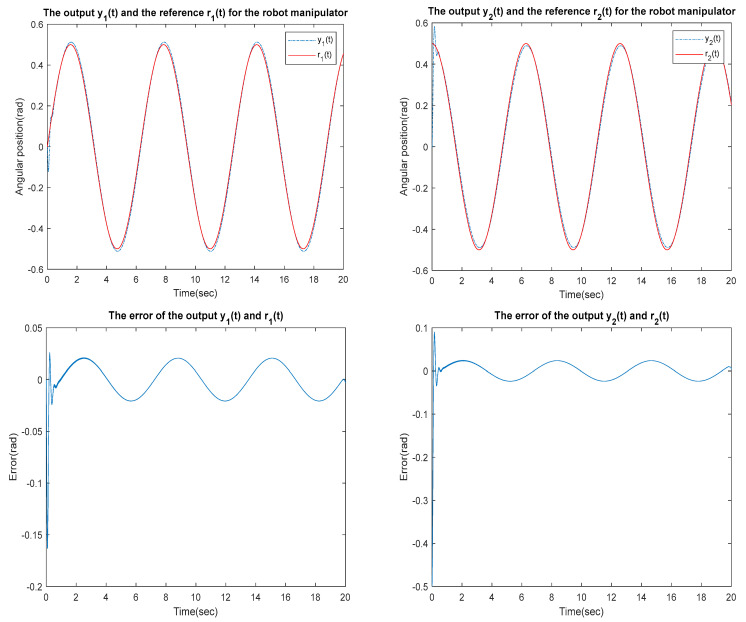
TYPE 1. Case 2: for the ability to suppress state delay.

**Figure 18:**
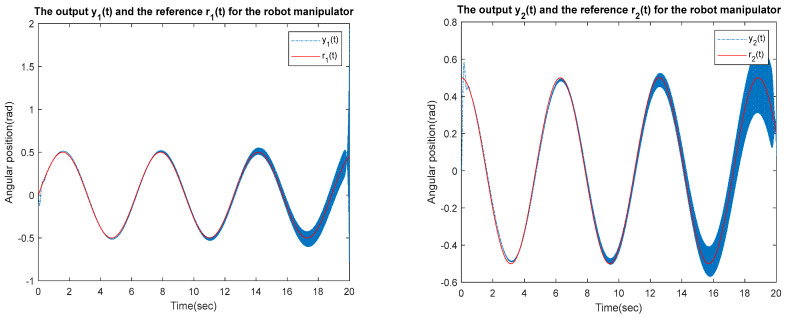

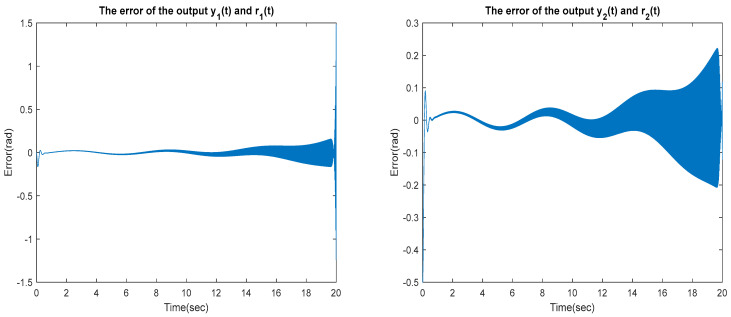
TYPE 1. Case 3: for the ability to suppress state delay.

**Figure 19:**
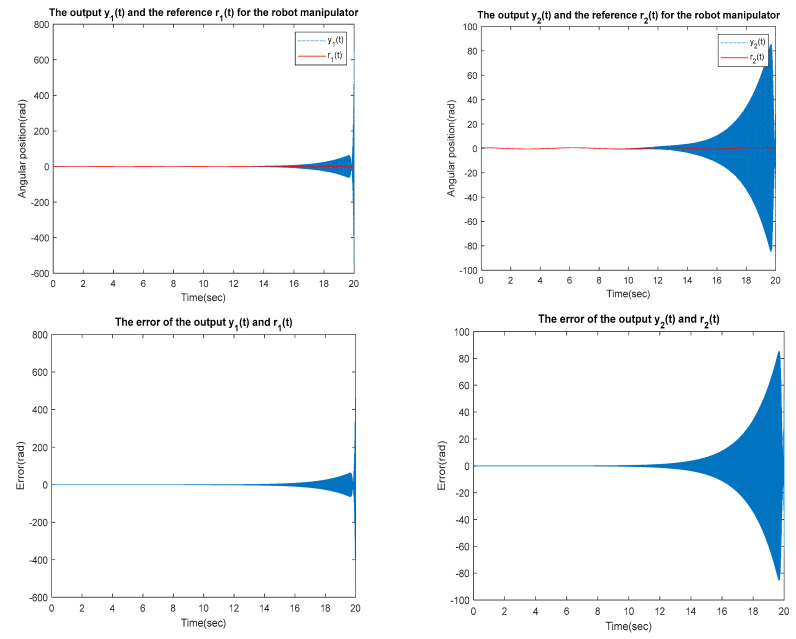
TYPE 1. Case 4: for the ability to suppress state delay.

**Figure 20:**
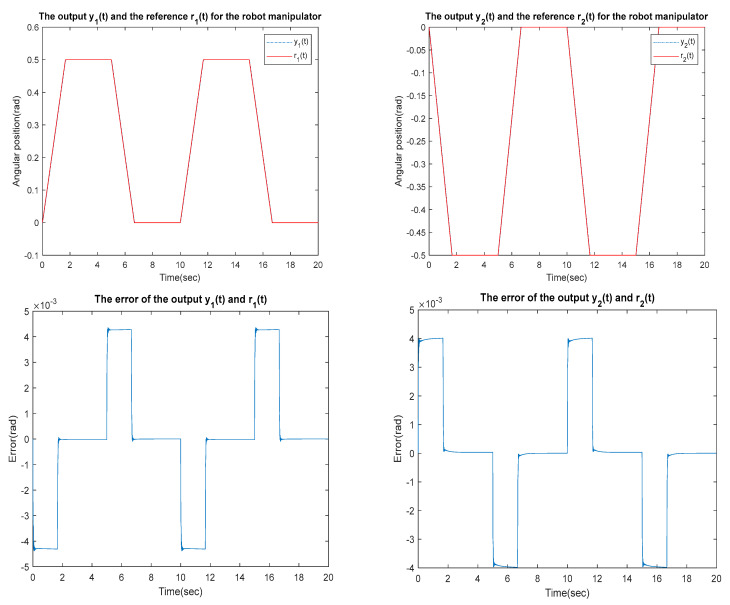
TYPE 2. Case 1: for the ability to suppress state delay.

**Figure 21:**
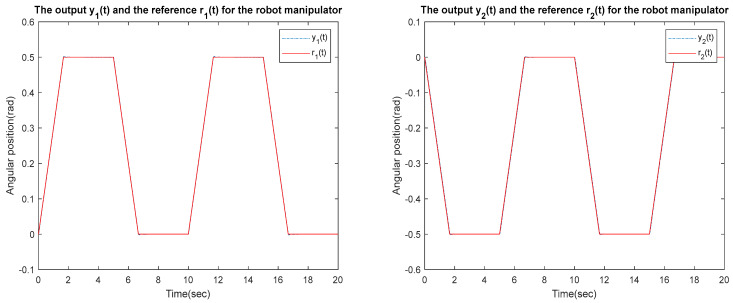

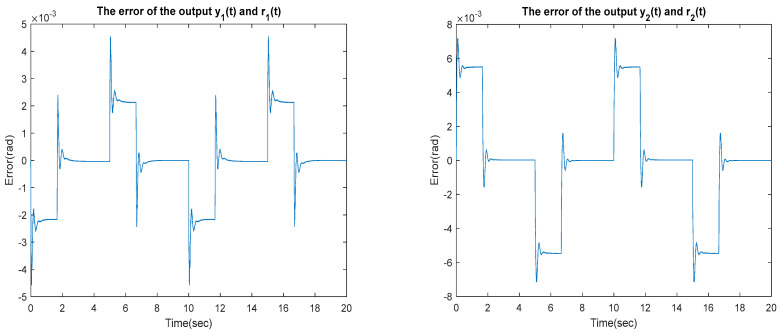
TYPE 2. Case 2: for the ability to suppress state delay.

**Figure 22:**
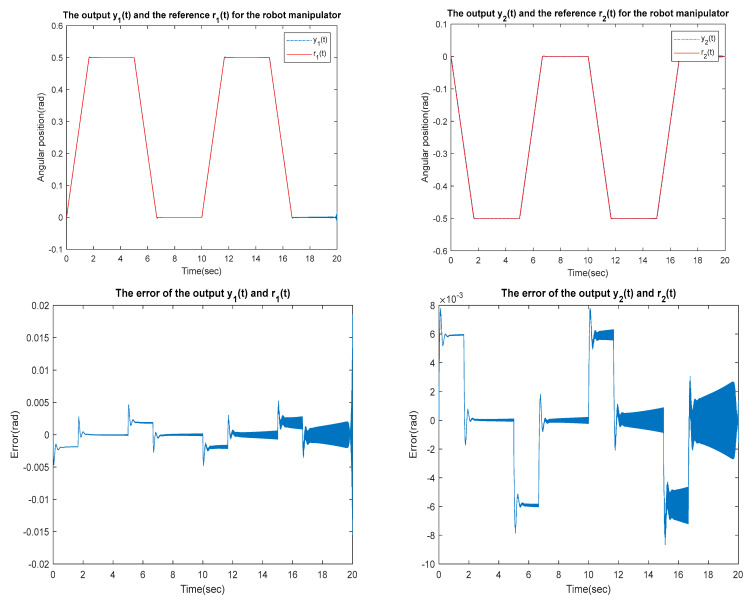
TYPE 2. Case 3: for the ability to suppress state delay.

**Figure 23:**
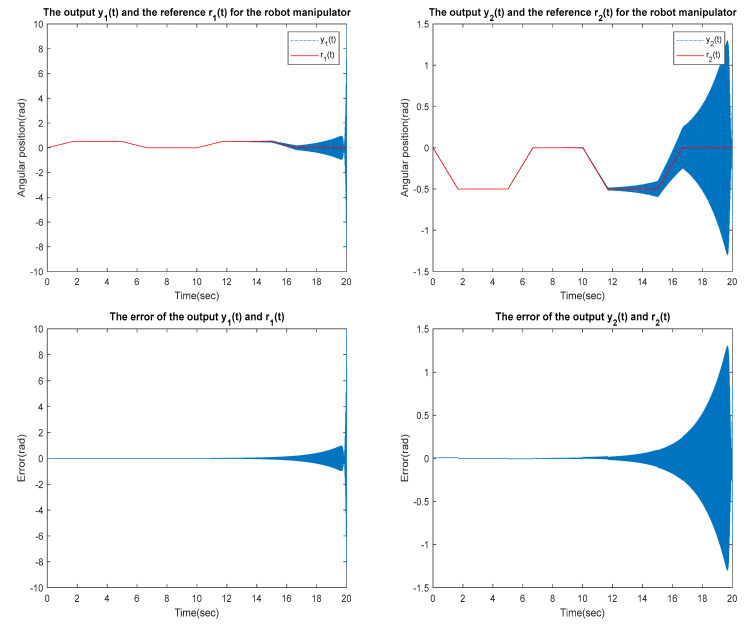
TYPE 2. Case 4: for the ability to suppress state delay.

We then consider two different reference inputs $r\left(t\right)={\left[\begin{array}{cc} r_{1} & r_{2} \end{array}\right]}^{T}$ for the identified equivalent linear system at the sampling time T=0.01 s with an input delay $\tau_{i}=0.5\times T$ and an output delay $\tau_{o}=0.3\times T$ for the optimal DR method presented in Section 4. To test whether the designed control can effectively suppress a state delay, we gradually increase the state delay $\tau_{s}=0\times T$ for two different reference inputs, i.e., Types 1 and 2. Figure 16, Figure 17, Figure 18, Figure 19, Figure 20, Figure 21, Figure 22 and Figure 23 show the ability of the control to suppress the Types 1 and 2 state delays. Table 1 summarises the results in Figure 16, Figure 17, Figure 18, Figure 19, Figure 20, Figure 21, Figure 22 and Figure 23, showing that the robot manipulator suppresses the state delay for approximately 2.6 s.

TYPE 1:

Case 1: $\tau_{i}=0.5\times T$, $\tau_{o}=0.3\times T$, $\tau_{s}=0\times T$

Case 2: $\tau_{i}=0.5\times T$, $\tau_{o}=0.3\times T$, $\tau_{s}=0.1\times T$

Case 3: $\tau_{i}=0.5\times T$, $\tau_{o}=0.3\times T$, $\tau_{s}=0.12\times T$

Case 4: $\tau_{i}=0.5\times T$, $\tau_{o}=0.3\times T$, $\tau_{s}=0.13\times T$

TYPE 2:

Case 1: $\tau_{i}=0.5\times T$, $\tau_{o}=0.3\times T$, $\tau_{s}=0\times T$

Case 2: $\tau_{i}=0.5\times T$, $\tau_{o}=0.3\times T$, $\tau_{s}=0.1\times T$

Case 3: $\tau_{i}=0.5\times T$, $\tau_{o}=0.3\times T$, $\tau_{s}=0.12\times T$

Case 4: $\tau_{i}=0.5\times T$, $\tau_{o}=0.3\times T$, $\tau_{s}=0.13\times T$

### 5.2. AI Training Results 

The next section shows the training results of the neural network. There are 325 training data points for the three subjects in the training and (bottle: 180; bowl and ball: 50). Noisy point cloud data are used for training to give a total of 595 training samples. There are 45 data points in a test set (bottle: 25; bowl and ball: 10). During the training period, the predicted output (joint angles or TCP) was standardization. The standardization range (Table 2) is set according to the working range of the arm.

Three models were trained for these three types of objects. The output for these models in Table 3 above is the UR5 arm joint angle that qbSoftHand uses to grasp the object. These angles are input into the UR5 control system and the arm is moved using the MoveJ command.

### 5.3. Real Machine Grasping

**Figure 24:**
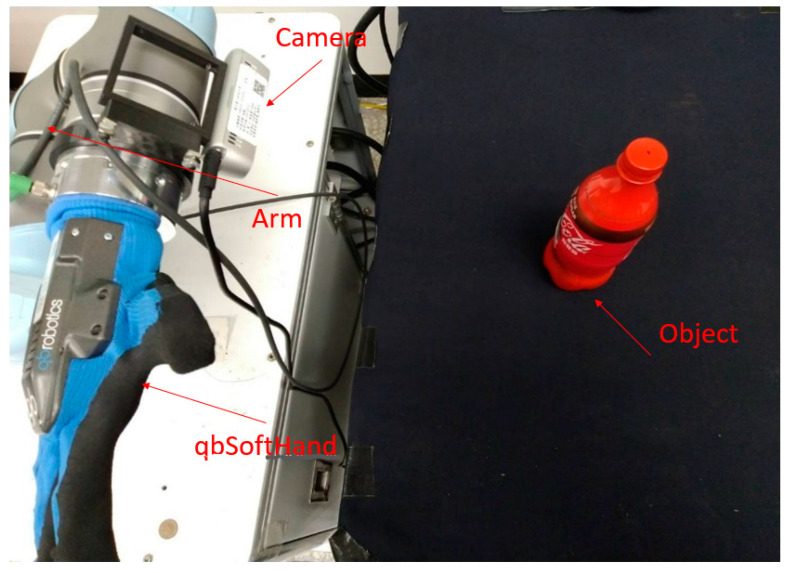
The UR5 arm observes the position of the object.

For the grasping test, the camera observes objects in the work space at an angle of about 45 degrees to the desktop (Figure 24).

#### 5.3.1. Five-Finger and Two-Finger Claw Gripping Experiment

**Figure 25:**
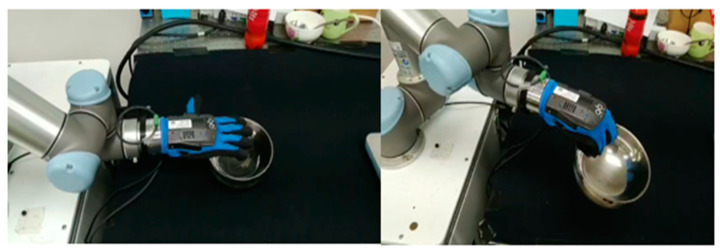
UR5 and qbSofthand grasping the bowl.

The experiment first tested the grip comparison between the two-finger and five-finger grippers (Figure 25). A network was trained using oriented bounding box (OBB) framework data [41] and a point cloud model using a two-finger gripper as a control group. Simultaneously, the results were compared with those of a study similar to [25]. The results are displayed in Table 4.

**Figure 26:**
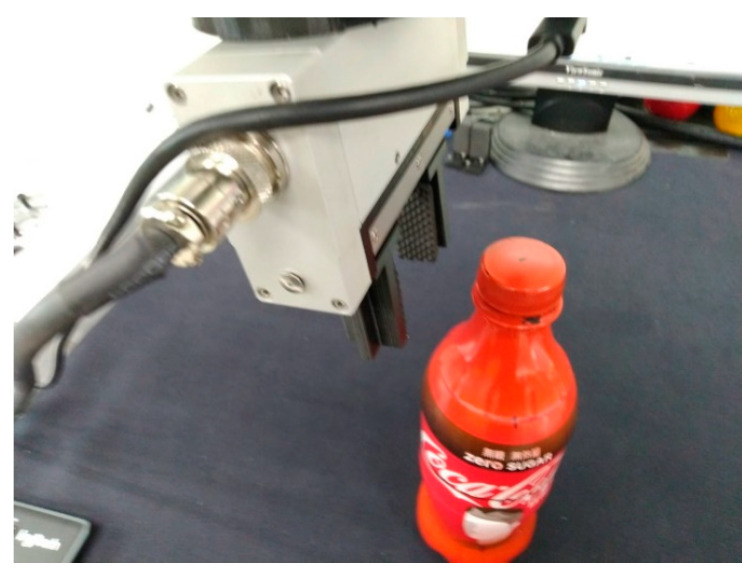
Failed attempt using a two-finger gripper.

According to the above test results, it can be observed that compared to the two fingers, the five-finger gripper achieved a success rate of more than 80% in the grip of various objects. The performance of the two-finger gripper (Figure 26), although it had a success rate of more than half, was much worse than that of the five-finger gripper. In fact, in most of the failed grips with the two-finger, the predicted position was very close to the object, but the object could not be grasped because the gripping area of the gripper was not wide enough and the force applied at the contact point. This demonstrates that even the five-finger grippers, which are only capable of open grip action, show a significant advantage over the two-finger on the hardware level. Reference [25] used a three-finger gripper and achieved at least an 80% success rate in both indoor and outdoor tests as in this study. Considering the larger number of samples they collected (570 in total), the similar success rate achieved in our study may be attributed to the adaptability of the five-finger gripper, in addition to the different experimental environment (clutter vs. single).

#### 5.3.2. Comparison of CNN and Min-Pnet Networks

The next section compares CNN networks, Min-Pnet networks designed with reference to the PointNet concept, and networks using OBB. Since the advantages of five fingers in hardware have been demonstrated in the previous section, it is shown that they can effectively compensate for the shortcomings of neural networks. In order to compare the differences in network architectures more clearly, the hardware was compared using a two-finger gripper. The results are shown in Table 5.

Compared to the CNN network, the results of Min-Pnet were significantly better in all aspects. Although the difference in the results on the bottles was not large (5%), the bowl and sports ball performed significantly better (24% and 20%), which may be due to the hardware limitation mentioned in the previous section, while for the OBB, except for the bowls with a larger successful area, the results were not very good. In the OBB experiment, it was found that objects that were too close or too far away were not successful, probably due to the angle of view and distance, so that the OBB frame point did not fully represent the size and position of the object.

#### 5.3.3. Out-of-Training Set Object Gripping

**Figure 27:**
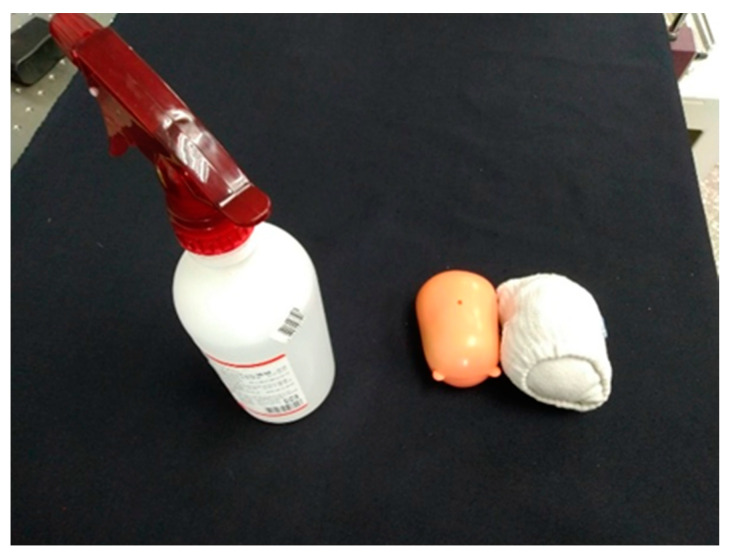
Out-of-training set object.

Then, we tested whether our network could produce a corresponding grip on an unknown object (see Figure 27) with a similar training set. The objects we tested were: an alcohol spray bottle (considered as bottle), a sock wrapped in a ball, and a small doll (considered as sport ball). The network included CNN, Min-Pnet and OBB, and the hardware was the same as in the previous section. The results are shown in Table 6.

Although the number of experiments is small, the success rate of Min-Pnet proves that the feature transformation architecture can indeed analyze point clouds more effectively, demonstrating its adaptability by still being able to predict effective grip positions when faced with objects that are similar but not identical to the training data. This also demonstrates the drawback of OBB’s difficulty in displaying asymmetric object features.

## 6. Conclusions

Simulation results in Section 5.1 are presented demonstrating effective optimal digital re-design control of a robot manipulator with a nonlinear delay. In particular, the proposed method can effectively control a state delay within the tolerable scope. 

In terms of gripper hardware comparison, although the qbSoftHand used in this study cannot grasp objects more flexibly due to hardware limitations, it can still grasp many objects that are difficult to grasp with the two-finger gripper, such as smooth bowls, balls, and bottles. A comparison with the two-finger gripper and the three-finger gripper of [25] demonstrates the advantage of the five-finger gripper on the hard surface.

In the case of objects outside the training set, the Min-Pnet achieved a success rate of more than 70% despite the small number of experiments, and after analysis, it was found that most of the gripping failures were due to hardware limitations. When testing the limits of the network, it is also proved that the features and poses of the objects cannot be fully expressed by using only the OBB framework.

Due to hardware and time constraints, it is not possible to conduct more complex experiments such as identifying and grasping multiple objects in a chaotic environment or testing the grasping of more objects in this paper. However, our research has partially demonstrated the advantages of the five-finger gripper and provided a simple and effective grasping system to automate the task of grasping objects.

There are many future research directions, such as using wireless communication to control the arm. The original data collection part can be accomplished in a virtual environment, or using 5G combined with AR for arm training, etc.

## Figures and Tables

**Table 1:** The ability of the robot manipulator to suppress state delays.

Reference Inputs	Delay Parameters	Max. Output Error (Rad)	Tolerable Delay Time of Manipulator
Type 1	$\tau_{i}=0.5\times T$ $,\;\tau_{o}=0.3\times T$ $,\;\tau_{s}=0.13\times T$	$6.48\times 10^{2}$	<2.6 s
Type 2	$\tau_{i}=0.5\times T$ $,\;\tau_{o}=0.3\times T$ $,\;\tau_{s}=0.13\times T$	9.886	<2.6 s

**Table 2:** Standardization range.

Range	Joint 1	Joint 2	Joint 3	Joint 4	Joint 5	Joint 6
Max (1)	−160°	−80°	140°	−10°	120°	0°
Min (0)	−220°	−120°	80°	−40°	40°	−50°
Range	X	Y	Z	α (Rx)	β (Ry)	γ (Rz)
Max (1)	1.5 m	1.5 m	1.5 m	1.5 m	1.5 m	1.5 m
Min (0)	−1.5 m	−1.5 m	−1.5 m	−1.5 m	−1.5 m	−1.5 m

**Table 3:** AI Model training results.

Model	Bowl Loss	Bowl Val-Loss	Ball Loss	Ball Val-Loss	Time Consumed
CNN	2.2469 × 10^−5^	0.0011	1.2599 × 10^−5^	1.7467 × 10^−4^	17 ms/epoch
Min-Pnet	1.4181 × 10^−4^	0.0011	5.3420 × 10^−5^	3.6437 × 10^−5^	215 ms/epoch
OBB	2.8651 × 10^−4^	0.0012	1.2412 × 10^−4^	2.4541 × 10^−4^	25 ms/epoch

**Table 4:** Bowl and ball gripping test results.

Object and Model	Grasping Success Rate
5-Fin CNN Bowl	90.0%
5-Fin CNN Ball	80.0%
5-finger CNN Bottle	80.0%
2-Fin CNN Bowl	58.82%
2-Fin CNN Ball	52.94%
2-finger CNN Bottle	64.15%
Indoor [25]	85%
Outdoor [25]	80%

**Table 5:** Test result of each network.

Object and Model	Grasping Success Rate
2-Fin Min-Pnet Bowl	82.60%
2-Fin Min-Pnet Ball	73.07%
2-finger Min-Pnet Bottle	69.01%
2-Fin CNN Bowl	58.82%
2-Fin CNN Ball	52.94%
2-finger CNN Bottle	64.15%
2-Fin OBB Bowl	60.00%
2-Fin OBB Ball	33.34%
2-Fin OBB Bottle	53.33%

**Table 6:** Out-of-training set object grabbing test.

Object and Model	Grasping Success Rate
2-Fin Min-Pnet Ballobj	75.00%
2-Fin CNN Ballobj	50.00%
2-Fin OBB Ballobj	25.00%
2-Fin Min-Pnet Bottleobj	73.33%
2-Fin CNN Bottleobj	60.00%
2-Fin OBB Bottleobj	53.33%

## Data Availability

Not applicable.
